# Spatial distribution characteristics and causes of public sports venues in China

**DOI:** 10.1038/s41598-023-42308-6

**Published:** 2023-09-12

**Authors:** Yueting Zhang, Yikeranmu Yi Ming, Bing Shi

**Affiliations:** Physical Education School, Shannxi Normal University, Xi’an, 710000 China

**Keywords:** Environmental social sciences, Sustainability

## Abstract

Public sports venues serve as a crucial material medium for human athletic activities. Research on their spatial distribution holds profound implications for optimizing spatial layout and promoting human sports endeavors. This paper takes interest points of sports venues in China as research samples, employing GIS spatial analysis tools, mathematical statistics, and Geo-detector as research methods. It systematically investigates the spatial distribution characteristics and causes of sports venues in China, aiming to provide insights and references for the optimization of spatial arrangement, sustainable development, and relevant policy formulation of sports venues in the country. The results indicate that: (1) In terms of overall distribution characteristics, sports venues in China present a multi-centric agglomeration in geographical space. (2) From a regional distribution perspective, the spatial layout of these venues reveals a pattern of “more in the south, less in the north,” “dense in the southeast, sparse in the northwest,” and a coastal strip distribution. (3) Regarding spatial correlation, the hot and cold spot partitions of sports venues largely align with the “Hu-Line”, with their spatial distribution tending towards a positively correlated pattern of high–high clustering and low–low clustering. (4) There is evident heterogeneity in factors affecting sports venues’ spatial distribution. The distribution results from the interactive coupling of multiple factors, where the interaction between any two factors offers stronger explanatory power for the spatial layout of the venues.

## Introduction

Public sports venues (hereinafter referred to as “sports venues”) refer to recreational facilities where the general public engages in sports competitions, training, fitness, entertainment, and other activities. In various countries and regions, these sports venues not only cater to the public’s fitness and entertainment needs but also play a crucial role in enhancing local image and contributing to high-quality urban development^1–3^. In the early twentieth century, the rapid diffusion of Western sports in China catalyzed the emergence of modern sports venues and infrastructures^4^. With the continuous improvement of people’s living standards, the increase in leisure time, and the heightened awareness of fitness, there has been a corresponding rise in the demand for spatial and infrastructural features of sports venues^5,6^. To meet the fitness needs of the population and improve the overall health standards^7^, the Chinese government has prioritized the construction of sports venues nationwide as a pivotal initiative in its endeavor to be a leading sports nation. However, with the burgeoning number of sports venues, challenges have arisen, including suboptimal spatial arrangements, underutilization of resources, and, consequently, long-term idleness, dereliction, operational inefficiencies, and prominent supply–demand discrepancies in many of these venues^8^.

Current research on sports venues has seen scholars exploring the topic from various angles. From a sociological perspective, topics include: terrorist attacks on sports venues^9^, naming patterns of these venues^10^, risks associated with sporting events held within them, the spirit of Olympic sports collaboration, and systematic risk management^11^. Economically, scholars have documented financing methods for sports venues^12^ and delved into audience-perceived risks, price perceptions, and continued consumption intentions^13^. In the field of management, research focuses on the performance management and operational evaluation of sports venues by establishing assessment criteria^14^. Analyses have been conducted on the opportunities and challenges sports venues face from macro and micro external environments to internal management^15^, how big data intelligence can enhance sports venue management^16^, and considerations on how to schedule matches efficiently across multiple venues^17^.

From a geographical perspective, however, research on sports venues remains somewhat limited. Bolz et al., using primary sources, traced the emergence of the public’s demand for leisure and sports venues, illustrating how sports and leisure spaces have been integrated into the national landscapes of Italy, Germany, and the UK^18^. Li et al. examined the differences between new urbanization and traditional urbanization in terms of county-level public sports venues, analyzing the current status and influencing factors of public sports venues in Chinese counties^19^. To date, only a handful of studies have analyzed the spatial distribution patterns of sports venues in China, with scholars often adopting a more micro-level case approach^20,21^.

Upon reviewing the aforementioned literature, it becomes evident that current academic discourse on sports venues is not comprehensive enough, with most scholars concentrating on a range of social issues arising post-construction of these facilities. However, meticulous exploration of the spatial distribution of sports venues prior to their construction, and research aimed at optimizing such distribution, should not be overlooked either. While a few researchers have delved into the spatial structure of sports venues, existing studies predominantly focus on case studies and qualitative descriptions at the provincial or city level. Quantitative multi-indicator analyses of the spatial distribution characteristics and causative factors of sports venues from a macro perspective have largely been neglected. This has, to an extent, hindered the strategic layout of sports venues by governments.

In light of this, our study focuses on sports venues across 31 provinces, cities, and autonomous regions in China (excluding Hong Kong, Macau, and Taiwan). By employing research methods such as kernel density estimation analysis, imbalance indices, and spatial auto-correlation analysis, we systematically unveil the spatial distribution characteristics of sports venues in China. Using geographic detectors, we dissect the spatial distribution of these venues to reveal underlying causative factors. We also analyze the current status of sports venues and put forth pertinent policy recommendations, aiming to promote the optimization and sustainable development of the spatial layout of these facilities.

**Figure 1:**
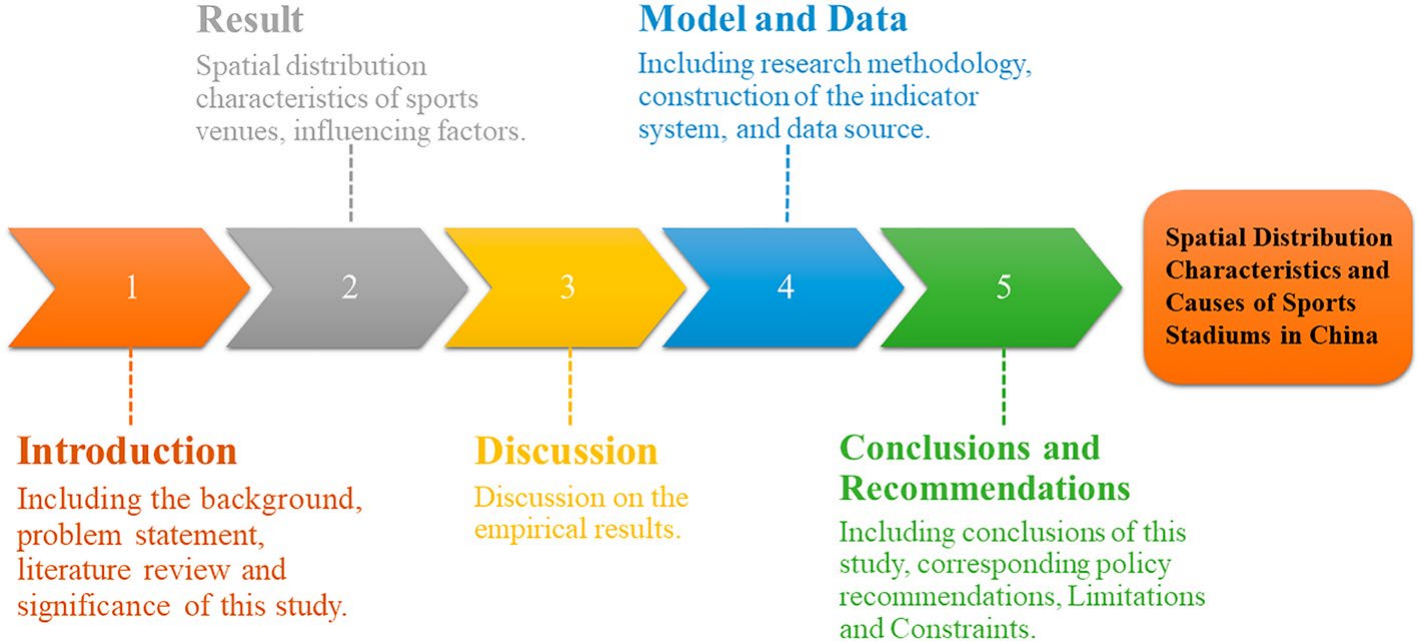
Flow chart of this study.

This study aims to address the following questions: What are the spatial distribution patterns and characteristics of sports venues? What is the nature of spatial relationships among sports venues across different regions and the clustering patterns within these areas? What spatial heterogeneity features result in this geographical phenomenon, and what are the underlying driving factors? Addressing these questions will provide international scholars with a comprehensive and in-depth understanding of the current spatial distribution and existing challenges of sports venues in China. Furthermore, the results of this study will offer theoretical insights for the sustainable development of China’s sports venues and the formulation of relevant policies. Additionally, it will present valuable lessons for the sustainable development of sports venues in other Asian countries or regions with similar cultural and economic contexts. The flowchart of this study can be seen in the figure below (Fig. 1).

## Results

### Spatial distribution characteristics of sports venues

#### Overall distribution characteristics

##### Spatial distribution equitability

**Figure 2:**
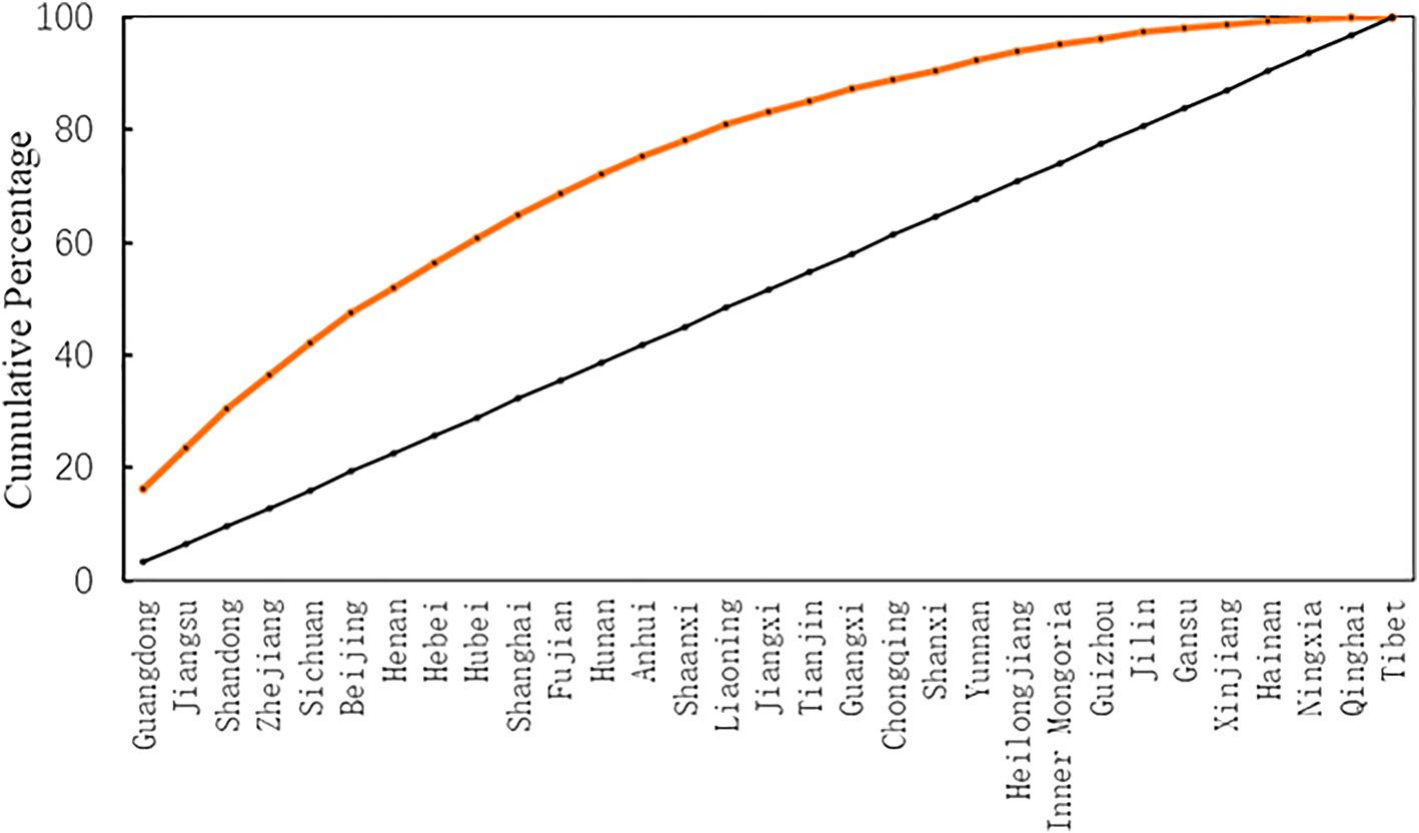
Lorentz curve of the Spatial distribution of national sports venues.

The imbalance index can reveal the degree of equitability in the distribution of sports venues within provinces and cities. By calculation, the imbalance index S is 0.47. Since S ranges between 0 and 1, it indicates that the distribution of sports venues among the provinces and cities is highly inequitable. Plotting the Lorenz curve (Fig. 2) reveals a pronounced convex trend on the curve. Provinces and cities such as Guangdong, Jiangsu, Shandong, Zhejiang, Sichuan, Beijing, Henan, Hebei, Hubei, and Shanghai account for nearly two-thirds of the total number of sports venues, while the remaining provinces and cities only make up one-third. This demonstrates that the spatial distribution of sports venues in China is highly uneven.

##### Kernel density estimation analysis

**Figure 3:**
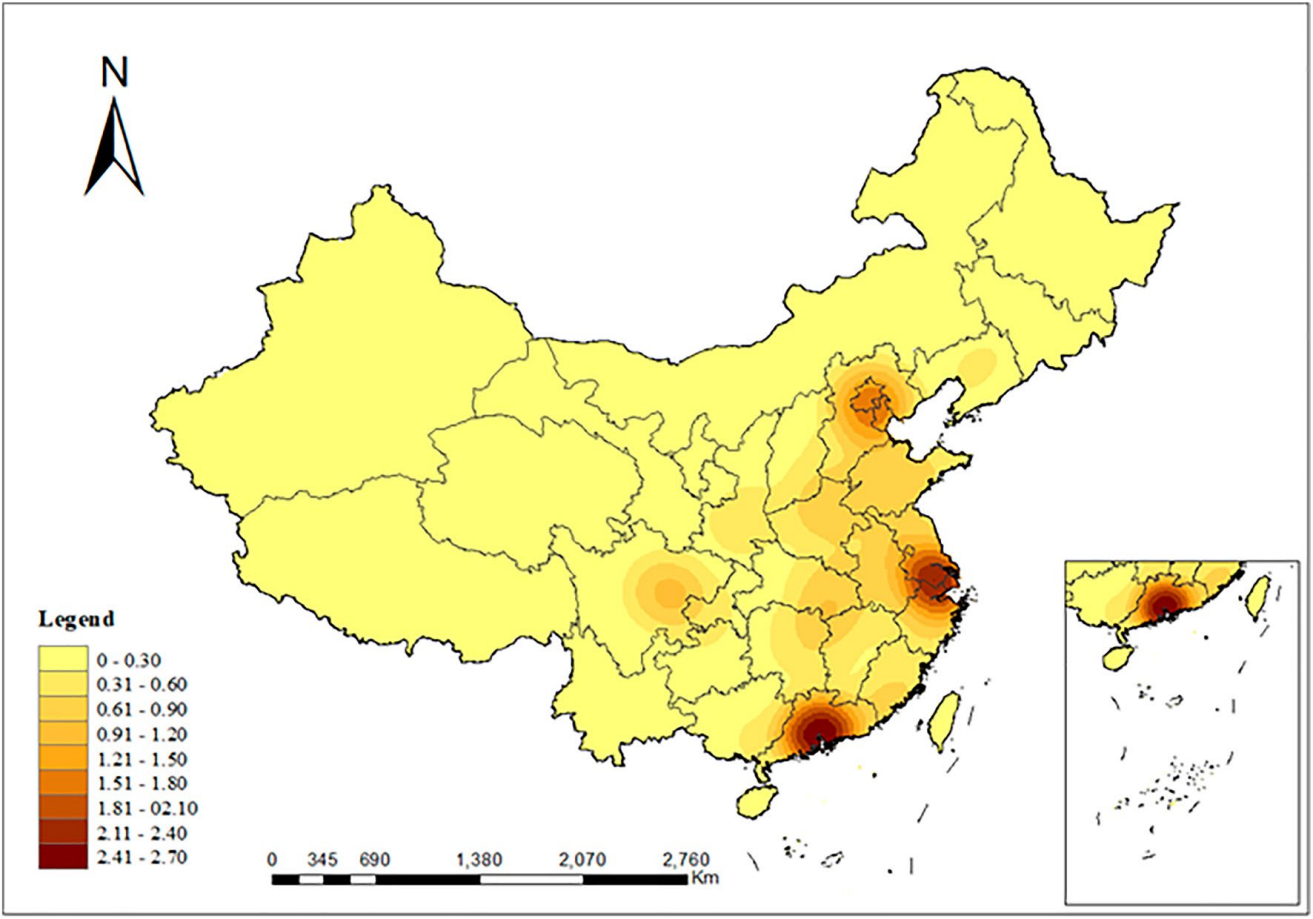
Distribution characteristics of core density of sports venues.

Kernel density estimation analysis provides a clearer representation of an element’s unit density within its surrounding neighborhood, compensating for the spatial distribution nuances that the nearest neighbor index and imbalance index may not intuitively reflect^22^. Based on the kernel density estimation formula (Fig. 3), sports venues have formed a high-density distribution pattern around major cities like Beijing, Shanghai, and Guangzhou, constituting 3 areas or 10% of the total. Intermediate high-density patterns are seen in areas like Tianjin, Shandong, Henan, Hebei, Jiangsu, Sichuan, Anhui, Hubei, Hunan, Zhejiang, and Fujian, accounting for 11 areas or 35%. Lesser density patterns are apparent in areas like Liaoning, Jilin, Shanxi, Shaanxi, Ningxia, Gansu, Jiangxi, Chongqing, Guizhou, Yunnan, and Guangxi, which also represent 11 areas or 35%. Low-density patterns manifest in areas such as Heilongjiang, Inner Mongolia, Gansu, Qinghai, Hainan, Xinjiang, and Tibet, covering 7 areas or 23% of the total. This highlights that sports venues in China exhibit a clustered spatial distribution characteristic.

#### Regional distribution characteristics

##### North–South distribution features

Using the Qinling Mountains and Huai River as the dividing line for northern and southern China. The northern region includes Beijing, Tianjin, Hebei, Shaanxi, Heilongjiang, Jilin, Liaoning, Inner Mongolia, Xinjiang, Tibet, Gansu, Qinghai, Ningxia, Shanxi, Henan, and Shandong. Sports venues in the northern region account for approximately 37% of the country’s total. The southern region encompasses Shanghai, Jiangsu, Anhui, Hubei, Sichuan, Chongqing, Yunnan, Guizhou, Hunan, Jiangxi, Zhejiang, Guangdong, Guangxi, Fujian, and Hainan. The sports venues in the southern region constitute about 63% of the national total. From this north–south perspective, the spatial distribution of sports venues in China presents a geographical trend of being more concentrated in the south and less so in the north.

##### The “Hu-Line”

**Figure 4:**
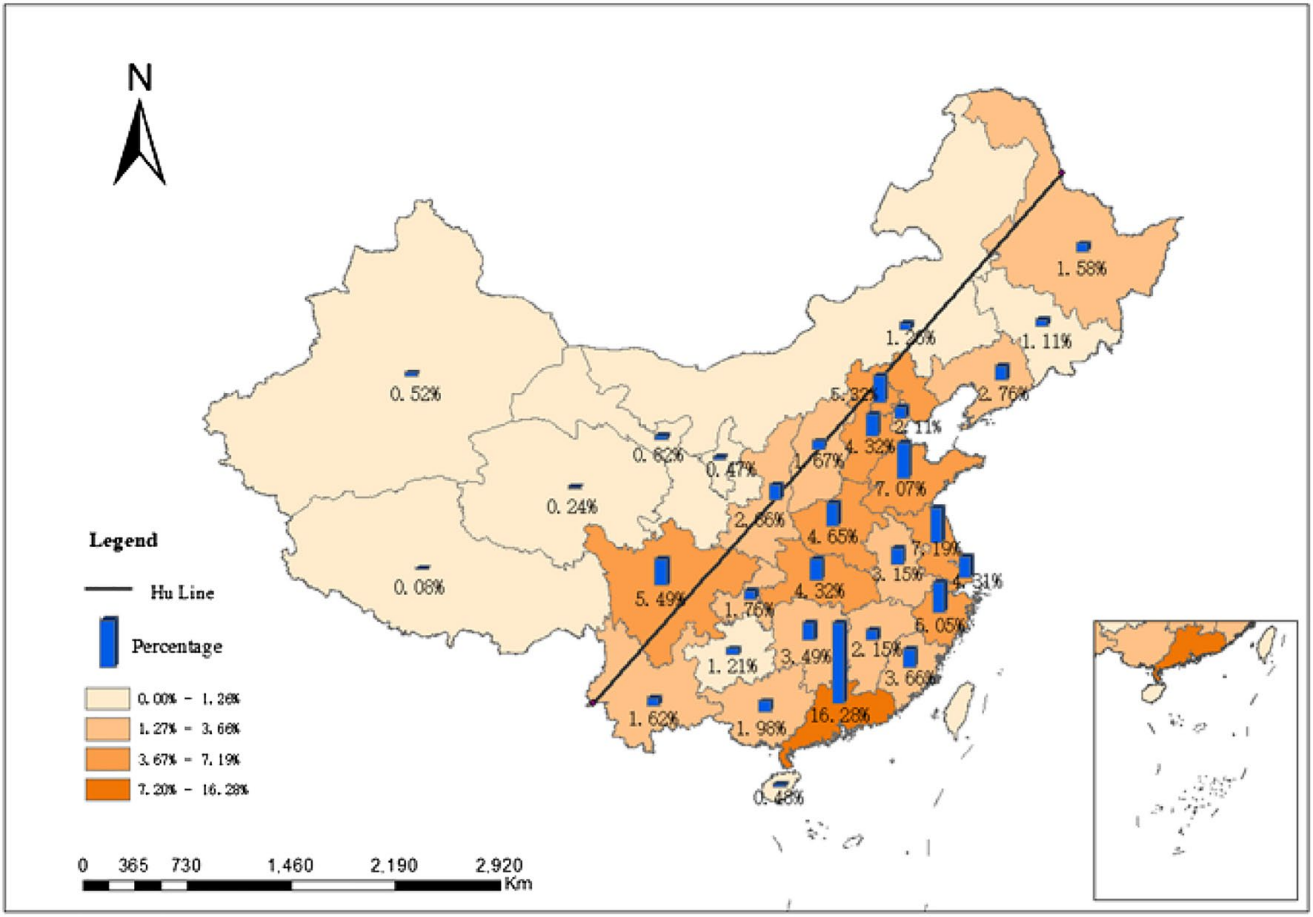
Distribution characteristics of sports venues on the provincial scale.

The “Hu-Line” serves as a significant demarcation of population density and economic development in China. It is one of the vital geographical discoveries reflecting the relationship between China’s population and its land^23^. This line also stands out as a notable boundary for the spatial distribution of sports venues in China. Observing from the perspective of the “Hu-Line” (as shown in Fig. 4), the spatial distribution of sports venues in China largely aligns with this line. The venues are primarily concentrated in the southeastern half of the “Hu-Line” area. The spatial distribution differences of sports venues can be vividly represented when comparing the southeastern side to the northwestern side of this line.

##### Distribution characteristics of the eight economic zones

From the perspective of socio-economic development, the spatial distribution of sports venues across the country can be segmented into eight distinct regions: (1) Northeastern Region, comprising Heilongjiang, Jilin, and Liaoning. (2) Northern Coastal Region, covering Beijing, Tianjin, Hebei, and Shandong. (3) Eastern Coastal Region, which includes Shanghai, Zhejiang, and Jiangsu. (4) Southern Coastal Region, encapsulating Fujian, Guangdong, and Hainan. (5) Middle Yellow River Region, consisting of Shaanxi, Shanxi, Henan, and Inner Mongolia. (6) Middle Yangtze River Region, which encompasses Hubei, Hunan, Jiangxi, and Anhui. (7) Southwestern Region, embracing Chongqing, Guangxi, Yunnan, Guizhou, and Sichuan. (8) Northwestern Region, taking in Gansu, Qinghai, Ningxia, Tibet, and Xinjiang.

**Figure 5:**
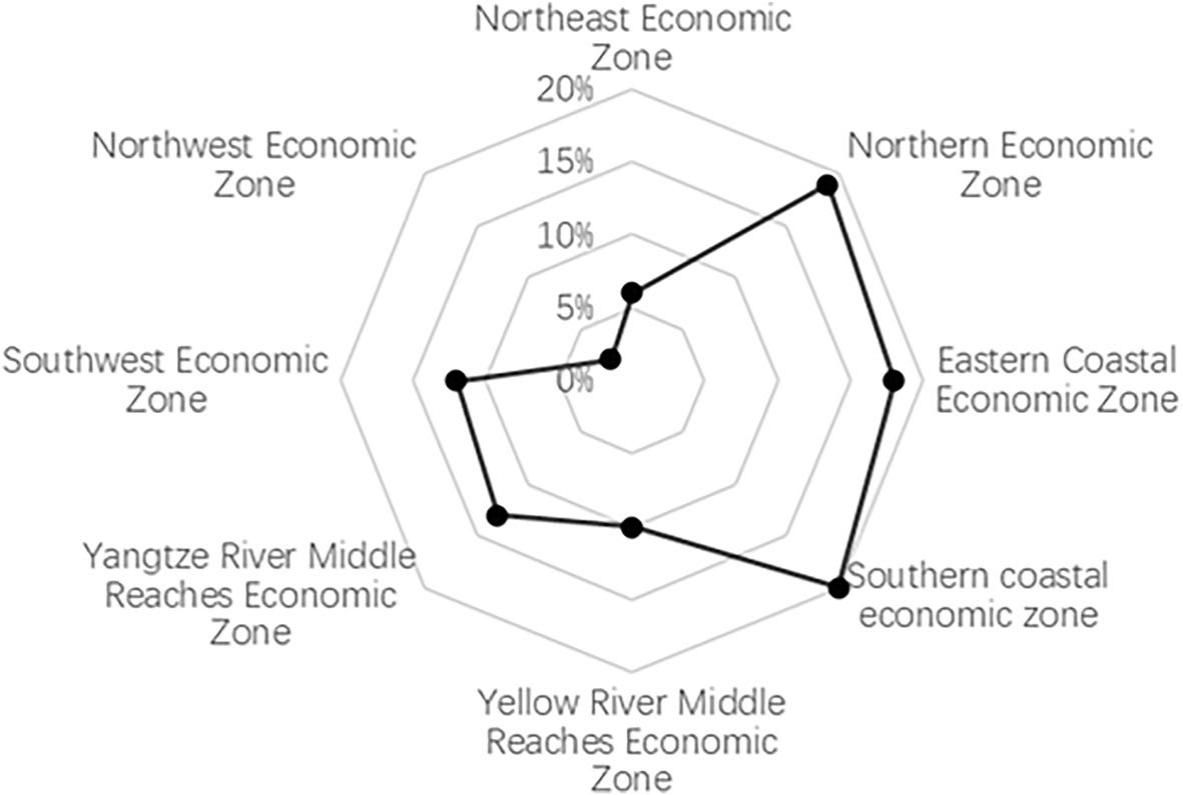
Distribution characteristics of National sports venues in eight major economic zones.

The proportional distribution of sports venues across these zones is as follows: Northeastern Economic Zone constitutes approximately 6%; Northern Coastal Economic Zone is around 19%; Eastern Coastal Economic Zone comprises about 18%; Southern Coastal Economic Zone represents close to 20%; Middle Yellow River Economic Zone holds approximately 10%; Middle Yangtze River Economic Zone stands at around 13%; Southwestern Economic Zone amounts to about 12%; and the Northwestern Economic Zone makes up roughly 2%. Analyzing these eight economic divisions, the distribution of sports venues is characterized by a gradual decrement, starting from the Northern Coastal Economic Zone, transitioning through the Eastern Coastal and Southern Coastal Economic Zones, and concluding in the Middle Yellow River, Middle Yangtze River, and Northwestern Inland Economic Zones. Specifically, the Southern Coastal Economic Zone holds the highest proportion, whereas the Northwestern Economic Zone contains the least, exhibiting a coastal belt-like distribution pattern (as depicted in Fig. 5).

#### Spatial correlation

##### Global spatial autocorrelation

Through the calculation using the Moran’s Index formula, a value of 0.17 > 0 was derived, with a *P*-value of 0. This indicates that, with a 99% confidence level in the significance test, we can decisively reject the null hypothesis that sports venues are distributed randomly. From this, it can be concluded that sports venues exhibit a significant spatial correlation, demonstrating a clustered distribution pattern.

##### Local spatial autocorrelation

**Figure 6:**
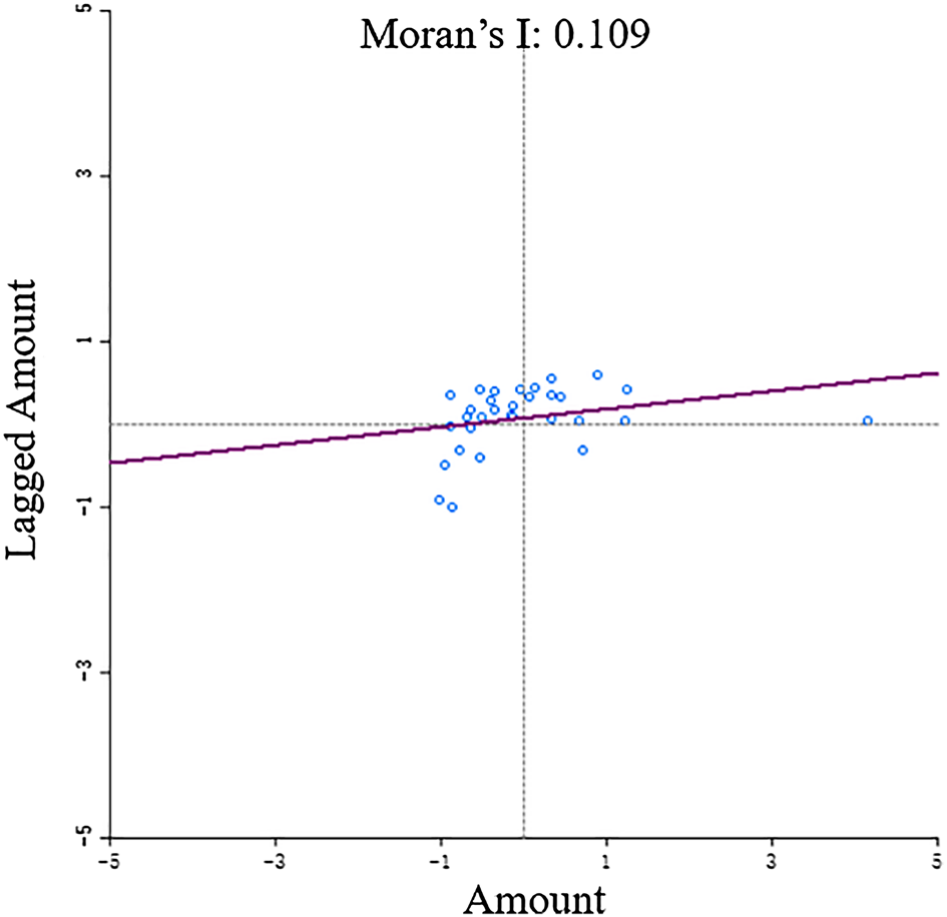
The Moran’ Scatter Diagram of distribution characteristics of national sports venues.

Utilizing GeoDa to establish spatial weights, the Moran scatter plot (Fig. 6) was constructed. Among the 31 provinces and municipalities: Regions falling into the first quadrant of the Moran scatter plot include: Guangdong, Fujian, Jiangsu, Shanghai, Zhejiang, Beijing, Shandong, Hebei, Henan, Hubei, and Hunan. Regions situated in the second quadrant of the Moran scatter plot comprise: Jilin, Liaoning, Tianjin, Shaanxi, Shanxi, Anhui, Jiangxi, Chongqing, Yunnan, Guangxi, Guizhou, and Hainan. Regions located in the third quadrant of the Moran scatter plot encompass: Heilongjiang, Inner Mongolia, Ningxia, Gansu, Qinghai, Xinjiang, and Tibet. The region in the fourth quadrant of the Moran scatter plot is: Sichuan. Upon comprehensive analysis of the 31 provinces and municipalities, 18 administrative regions, representing 58%, are situated in the first and third quadrants of the Moran scatter plot. Meanwhile, 13 administrative regions, accounting for 42%, are found in the second and fourth quadrants. Specifically, around 35% of administrative regions are in the first quadrant, and approximately 39% are in the second quadrant. This suggests that the spatial distribution of sports venues exhibits significant geographical disparities, reflecting a spatially positive correlation pattern where sports venues tend to cluster in areas of both high-high concentration and low-low concentration.

### Exploration of causal factors for the spatial distribution of sports venues

#### Detection of singular influence factors

To delve deeper into the determinants of the spatial distribution of sports venues in China, we first discretized each influencing factor. Subsequently, these discretized factors were imported into the Geo-Detector to compute their explanatory power over the geographical distribution of sports venues (Table 1).

**Table 1:** Correlation analysis of spatial distribution and influencing factors of sports venues in China.

Influencing factors	Impact factors	q value	*p* value	Sorting
Socio-economic factors	End-of-year Total Population (10,000 people)	0.1491	0.00	6
	Total GDP (10,000 RMB)	0.4478	0.00	1
	Per Capita GDP (RMB)	0.1022	0.00	7
	Urbanization Rate (%)	0.0754	0.00	9
	Disposable Income of Urban Residents (RMB)	0.3812	0.00	2
	Disposable Income of Rural Residents (RMB)	0.2335	0.00	4
	Number of Public Buses per 10,000 People (vehicles)	0.1629	0.00	5
	Road Network Density (km/100 km^2^)	0.0981	0.00	8
Natural environment	Altitude (m)	0.0625	0.00	10
	Slope of Terrain (°)	0.0135	0.05	11
	Temperature (°C)	0.3061	0.00	3

##### Socio-economic factors

Socio-economic determinants serve as the prerequisites for the development of sports venues, directing their spatial distribution. The economic radiative power is an integral and vibrant component of an area’s infrastructure development^24,25^. Generally, regions with advanced economic growth have distinctive advantages in areas such as financial resources, technology, talent, services, markets, and infrastructure. From a socio-economic perspective, the q-values for specific indicators such as total population, total GDP, GDP per capita, urbanization rate, disposable income of urban residents, disposable income of rural residents, buses per 10,000 people, and road network density are respectively: 0.1491, 0.4478, 0.1022, 0.0754, 0.3812, 0.2335, 0.1629, and 0.0987. The total GDP and disposable income of urban residents stand out as the most significant influencers, demonstrating robust explanatory power over the spatial distribution of sports venues. Following these, rural disposable income, buses per 10,000 people, year-end total population, GDP per capita, road network density, and urbanization rate possess relatively weaker dependencies regarding sports venue distribution. As sports venues primarily serve as community recreational centers, they tend to align with the economic spatial layout. The greater the economic development, the more the number of sports venues, indicating a significant positive correlation between the scale of sports venues and the level of economic growth. In China, sports venues prominently cluster in economically advanced areas such as Beijing, Shanghai, Guangdong, Jiangsu, Zhejiang, and Fujian. This distribution can be attributed to factors such as the principal investors in sports venues, the hosting of major sports events, and historical influences. Firstly, local government departments are the primary investors in sports venues across provinces and cities, with government financial appropriations being the primary source of funding. Secondly, certain regions in China have constructed numerous sports venues in conjunction with hosting significant sports events. For instance, the first nine National Games of China alternated between Beijing, Shanghai, and Guangdong. Moreover, Beijing hosted events like the 29th Summer Olympics and the 24th Winter Olympics. Thirdly, prior to the liberation, the general distribution of venues during that period was influenced by factors like the influx of overseas Chinese, the spread of modern sports, and venue development policies, which predominantly situated these venues in eastern coastal cities and some central cities^26^. Furthermore, regions like Beijing-Tianjin-Hebei, Jiangsu-Zhejiang-Shanghai, and areas surrounding the Yangtze River, with their extensive transportation coverage and high accessibility, facilitate the layout of sports venues, promoting their development to a certain extent. Conversely, areas in the western regions of China like Xinjiang, Tibet, and Yunnan face challenges due to their natural geographical environment, lagging transportation development, and reduced accessibility, which can inhibit the construction of sports venues. Generally, in areas with convenient transportation, not only can people reduce the time–cost of reaching sports venues, but they can also compensate to some extent for environmental disadvantages, enhancing the utility of sports venues. In essence, the more economically developed a region, the greater the number of sports venues. The agglomeration effect of economically prosperous areas on their surrounding regions surpasses their radiative influence, and a significant “Matthew effect” is evident between economically advanced areas and those with moderate economic levels.

##### Natural environmental factors

Natural environmental determinants serve as foundational elements in the development of sports venues, acting as the “cornerstone” for their establishment. Typically, the site selection for sports facilities necessitates consideration of topography and climatic conditions. Sports venues are predominantly located in regions with relatively flat terrains and favorable climatic conditions^25,27^, as such locations can decrease the construction costs and enhance the utilization rate of these facilities. Among the natural resource-driven factors, the influencing indicators, ranked in descending order of significance, are temperature (0.3061), altitude (0.0625), and slope (0.0135), with temperature emerging as the predominant factor. From a geographical perspective, the southern regions of China are primarily influenced by the subtropical monsoon climate, characterized by abundant precipitation, humid air, and high vegetation coverage. These conditions correlate with a higher number of sports venues. In contrast, northern regions, influenced by temperate monsoon climates, experience less precipitation, drier conditions, and have a comparatively lower vegetation cover, resulting in fewer sports venues. Analyzing China’s topography, which is generally categorized into three significant tiers: the first and second tiers are characterized by higher altitudes and sparser populations, and consequently, host fewer sports venues. The third tier, primarily comprising plains, mostly has altitudes below 500 m, presenting relatively flat terrains. With its higher population density, this tier displays a more concentrated spatial distribution of sports venues. Elevation plays a pivotal role in the construction of sports facilities. As the altitude increases, the number of sports venues decreases, and their spatial distribution becomes more sparse. Conversely, lower altitudes witness a larger number of sports venues with a denser spatial distribution. Building sports venues in regions with higher altitudes and steeper slopes involves higher costs and greater challenges, inherently constraining the development of such facilities in these areas.

#### Interaction factor analysis

**Figure 7:**
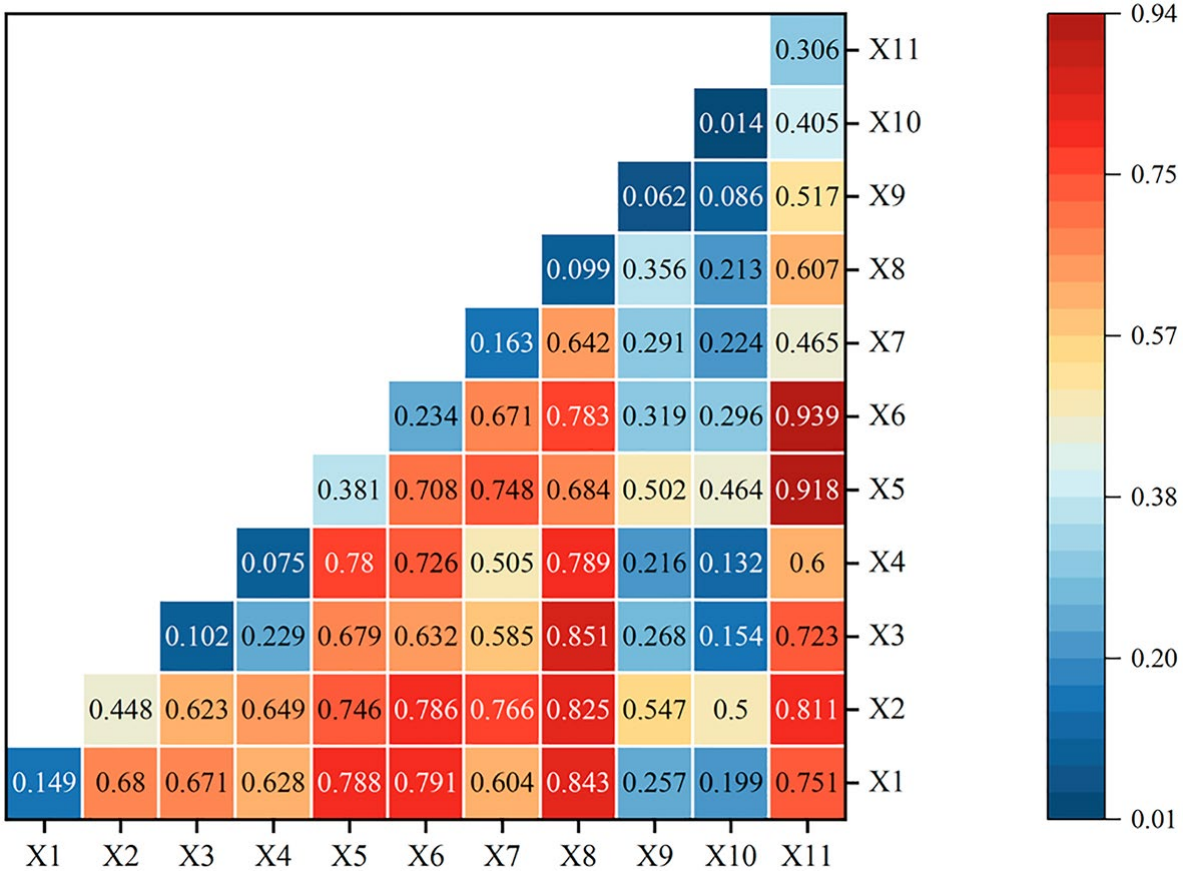
Detection results of interaction factors for spatial distribution of sports venues.

Exploring the interactive effects among individual influencing factors, findings derived from the interaction detection results (Fig. 7) reveal that interactions between factors consistently demonstrated enhanced relationships. These interactions can be categorized into two types: bilateral enhancement and nonlinear enhancement. No indications of bilateral nonlinear weakening, unilateral nonlinear weakening, or independent relationships were observed. This suggests that any interaction between two of the 11 influencing factors will further enhance the explanatory power regarding the spatial distribution of sports venues. In essence, the spatial distribution of these venues is the result of the intricate interplay of multiple factors.

There exists variability in the explanatory power of interactive effects among different factors. Overall, the interaction of the total GDP factor (X2) and urban resident disposable income factor (X5) with other influencing factors led to a notable increase in their influence, predominantly displaying bilateral nonlinear enhancement. Specifically: Interactions between total GDP and other influencing factors resulted in q-values consistently above 0.50. Notably, the interaction between the total GDP factor and road network density factor exhibited the highest q-value, with an explanatory power of approximately 83%. Interactions between urban resident disposable income and other influencing factors yielded q-values consistently above 0.40. Among these, the interaction between urban resident disposable income and temperature had the highest q-value, peaking at 0.918. This provides further affirmation that within the socio-economic factors, both the total GDP and urban resident disposable income factors are primary determinants influencing the spatial distribution of sports venues. Their interactive effects with other factors can adeptly elucidate the spatial distribution patterns of sports venues. Zooming in on specifics, interactions between the road network density factor and other influencing factors consistently showed bilateral nonlinear enhancement. For instance, post-interaction q-values of road network density with factors like year-end total population, total GDP, per capita GDP, and temperature were considerably higher than the standalone q-value for road network density. This suggests that even if a singular influencing factor might have a weaker q-value, its explanatory power post-interaction might not necessarily be inferior to factors with inherently higher q-values. In conclusion, the interactive effects between dual factors offer a more robust explanatory power regarding the spatial distribution characteristics of sports venues than singular influencing factors. The spatial distribution patterns of sports venues are thus shaped by the compounded interplay of multiple influencing factors.

## Discussion

In this study, we delved into the spatial distribution characteristics of sports venues in China, unveiling distinct patterns such as multi-centric clustering, a greater prevalence in the south compared to the north, and a concentrated distribution along the coastal regions. To a certain extent, these patterns mirror the regional developmental disparities and urbanization levels within China. Existing literature posits that resource allocation efficiency in the eastern parts of China surpasses that in other regions, leading to a pronounced spatial imbalance in the distribution efficiency of sports venues^28^. Our findings align with this observation. Previous studies, adopting a polarization theory perspective, differentiated China into its eastern, central, and western regions to analyze the allocation efficiency of sports venues. In contrast, our approach leveraged POI (Point of Interest) data, dissecting China from a north–south axis, dichotomizing it further along the “Hu-Line” into southeast and northwest segments, and additionally considering the spatial distribution across the eight major economic zones. This comprehensive approach provides a nuanced understanding of the spatial distribution of sports venues in the country.

In this study, we investigated the factors influencing the spatial distribution of sports venues in China. Our findings highlight the undeniable importance of socio-economic factors in the distribution of these venues. Specifically, the total GDP and urban residents’ disposable income indicators exhibit a strong explanatory power for the spatial distribution of sports venues, suggesting a tight correlation between economic development levels and the spread of these facilities. This perspective is bolstered by extant research^29^. Some studies indicate that the future layout of sports venues should thoroughly account for areas expecting significant population growth to ensure timely provision of sports facilities^30^. This affirms the significant influence of population size on the spatial distribution of sports venues. However, while these studies focus on the characteristics and utilization rates of users in facility layout, our research emphasizes a more comprehensive consideration of multiple factors. Yet, we did not delve deeply into matching the spatial distribution of sports venues with the specific demands of the local population, an avenue that warrants further exploration in subsequent research. From a macro perspective, there’s an observable correlation between the distribution of sports venues and a region’s population and economic levels. For instance, in economically developed regions with smaller populations, the number and quality of sports facilities tend to be superior. Conversely, in densely populated but economically underdeveloped areas, there is often a paucity of sports venues and facilities^26^. This phenomenon could be linked to local government fiscal input, residents’ ability to consume sports, and demand patterns. Our findings underscore the pivotal role of transportation conditions in shaping the spatial distribution pattern of sports venues^31^. Moreover, other driving factors influencing the spatial distribution of sports venues are of significance, resonating with existing research findings^32,33^. Compared to existing research^19^, our investigation pays closer attention to the impacts of socio-economic and natural environmental factors on sports venue distribution. Yet, we didn’t quantify the influence of governmental policies or compare it with other impacting factors. Their findings indicate that the government plays an instrumental role in the construction and spatial optimization of sports venues.

Existing research^34^, grounded in theoretical insights, has delved into the current status of public sports facilities in Jinan’s central urban area. This research posits that in the layout of public sports facilities across various levels, it is imperative to consider factors such as urban public transportation, urban management systems, urban public functions, and the rational service radius of public sports facilities. Both our study and this aforementioned research focus on the planning, layout, and distribution of sports facilities, emphasizing the necessity to factor in a myriad of elements during the planning process. However, their study leans more towards urban planning and layout, while our research is more centered on spatial distribution and the influencing factors therein. These distinctions might arise from differences in research objectives, backgrounds, and methodologies adopted by the respective researchers.

## Methods

### Research methodology

#### The imbalance index

The Imbalance Index can be employed to analyze the distribution equilibrium of sports venues across various provinces and cities. In this study, we utilized the method of calculating the concentration index from the Lorenz curve to reflect the uniformity of the distribution of the object of study across different regions^35^. The formula for calculation is as follows:

1 $$ S = \mathop \sum \limits_{i = 1}^{n} Y_{i} - 50n + 1/100 \times n - 50n + 1{ } $$

where S is the imbalance index; n is the number of provinces (cities, autonomous regions); Y_i_ is the cumulative percentage of the i-th order of the proportion of public sports venues in each province (city, autonomous region) to the total number of public sports venues in the country, sorted from largest to smallest. The value of the imbalance index S ranges from 0 to 1. If S = 0, it means that public sports venues are evenly distributed among different provinces (cities, autonomous regions); if S = 1, it means that public sports venues are all concentrated in one region; the larger the value of S, the more uneven the distribution of public sports venues.

#### Kernel density estimation analysis

Kernel Density Estimation is among the most prevalent methods for analyzing the distribution of point features^36^. It is employed to study the spatial density distribution of point features within a region, revealing the spatial distribution patterns and characteristics of the features. A higher kernel density value indicates a more concentrated distribution of points, while a lower value suggests dispersion^37,38^. Its computational formula is as follows:

2 $$ f_{h} \left( x \right) = \frac{1}{nh}\mathop \sum \limits_{i = 1}^{n} \left( {\frac{{x - x_{i} }}{h}} \right){ } $$

where: ${f}_{h}\left(x\right)$ represents the kernel density function, $x-{x}_{i}$ indicates the distance between x and ${x}_{i}$, h represents the width and must be greater than 0. The larger the value of ${f}_{h}\left(x\right)$, the denser the distribution of public sports venues.

#### Spatial autocorrelation analysis

Spatial Autocorrelation Analysis reflects the degree of correlation between a geographic phenomenon or a specific attribute value in one regional unit and the same phenomenon or attribute value in neighboring units^39^. Global spatial autocorrelation is utilized to determine the spatial association of sports venues within the national region, employing the $Moran{\text{'}}s \;I$ to measure the relationship between neighboring spatial distribution objects and their attribute values^40,41^. Its computational formula is as follows:

3 $$ Moran{\text{'}}s \;I = \frac{n}{{S_{0} }} \cdot \mathop \sum \limits_{i}^{n} \mathop \sum \limits_{j = 1}^{n} w_{ij} \left( {x_{i} - \overline{x}} \right)\left( {x_{j} - \overline{x}} \right)/\mathop \sum \limits_{i}^{n} \left( {x_{i} - \overline{x}} \right)^{2} $$

In the formula: n represents the number of spatial units; ${w}_{ij}$ represents the spatial weight matrix; ${x}_{i}$ and ${x}_{j}$ represent the observed values; $\overline{x }$ represents the mean. The value of $Moran{\text{'}}s \;I$ can be used to determine the degree of clustering of the sporting venues, with a value between − 1 and 1. If $I$ > 0, it indicates positive spatial correlation in the data, which means that the sporting venues have a clustered distribution. If $I$ < 0, it indicates negative spatial correlation in the data, which means that the sporting venues have a uniform distribution. If $I$ = 0, it means that the sporting venues have a random distribution.

Global spatial autocorrelation only reveals whether there is a spatial correlation in sports venues on a global scale, but it has limitations in reflecting local spatial correlations. Therefore, this study employs the Moran Scatterplot to further discern the degree of spatial clustering among sports venues in different regions. The analysis utilizes $\mathrm{Anselin }Local Mora{n}^{\mathrm{^{\prime}}}I$, and its computational formula is as follows^42^:

4 $$ Local\;Moran^{\prime}I = \frac{{x_{i} - \overline{x}}}{{S^{2} }}\mathop \sum \limits_{j = 1}^{n} w_{ij} \left( {x_{i} - \overline{x}} \right){ } $$

5 $$ S^{2} = \frac{{\mathop \sum \nolimits_{j = 1,j \ne 1}^{n} \left( {x_{i} - \overline{x}} \right)^{2} }}{n - 1} - \overline{x}^{2} $$

In the formula, ${S}^{2}$ refers to the variance of the observation values; $Local Mora{n}^{\mathrm{^{\prime}}}I$ the spatial autocorrelation analysis has four clustering relationships: High–High clustering (HH, first quadrant), Low–High clustering (LH, second quadrant), Low-Low clustering (LL, third quadrant), and High-Low clustering (HL, fourth quadrant). High-High clustering and Low-Low clustering indicate a positive spatial correlation between the public sports venues in a certain province or city and the surrounding provinces or cities, while Low–High clustering and High-Low clustering are the opposite.

#### Geographic detector

The Geographic Detector is a statistical method designed to detect and utilize geographical spatial distribution characteristics. It aims to uncover the underlying driving factors behind the spatial distribution of sports venues^43^. This method has been widely applied in both natural and social environments^44,45^.

1. Single Factor Detection: Factor detection can identify the explanatory power of each driving factor affecting the spatial distribution of sports venues. The computational model is as follows^46^:
  6 $$ {\text{q}} = 1 - \frac{{\mathop \sum \nolimits_{{{\text{h}} = 1}}^{{\text{L}}} {\text{N}}_{{\text{h}}} \sigma_{{\text{h}}}^{2} }}{{{\text{N}}\sigma^{2} }} = 1 - \frac{{{\text{SSW}}}}{{{\text{SST}}}} $$
  7 $$ SSW = \mathop \sum \limits_{h = 1}^{L} N_{h} \sigma_{h}^{2} ,SST = N\sigma^{2} $$
  In the formula, L represents the number of layers of the influence factor, N_h_ and N represent the number of samples in layer h and the whole respectively, ${\sigma }_{h}^{2}$ and ${\sigma }^{2}$ respectively represent the variance of layer h and the whole, SSW and SST represent the within-layer variance and the total variance respectively, and q represents the interpretive power of each index on the spatial distribution of public sports venues in our country, with a value range of [0,1]. The larger the q value, the greater the impact of the index on the spatial distribution of public sports venues.
2. Interactive Factor Detection: Different factors can have varying logical connections, either strong or weak. To further discern whether these factors, in interaction, increase or decrease their explanatory power on the spatial distribution of sports venues, this study employs the interaction detection method within the Geo-Detector to explore the interactive effects of 11 factors. Through comparing the interactions among factors, the influence of any two factors on the spatial distribution of sports venues can be determined. There are five types of interactions as follows: ① if q(X1 ∩ X2) < Min[q(X1),q(X2)], then the two factors are nonlinearly weakened; ② if Min[q(X1),q(X2)] < q(X1 ∩ X2) < Max[q(X1),q(X2)], then the single factor is nonlinearly weakened; ③ if q(X1 ∩ X2) > Max[q(X1),q(X2)], then the two factors are strengthened; ④ if q(X1 ∩ X2) = q(X1) + q(X2), then the two factors are independent; ⑤ if q(X1 ∩ X2) > q(X1) + q(X2), then the two factors are nonlinearly strengthened.

### Indicator selection and data source

#### Construction of the indicator system

In this study, adhering to the principles of scientific rigor, representativeness, and reliability, we drew inspiration from relevant research outcomes^47,48^. Taking into account the essential conditions that should be present for the establishment of sports venues, we constructed an indicator system affecting the spatial layout of sports venues, comprising 11 indicators from two dimensions: socio-economic and natural environment. (1) Socio-economic Dimension: The number of inhabitants, socio-economic development level, urbanization rate, and standard of living are foundational to the spatial distribution of sports facilities. Thus, we selected Total Population (X1), Total GDP (X2), Per Capita GDP (X3), Urbanization Rate (X4), Disposable Income of Urban Residents (X5), and Disposable Income of Rural Residents (X6). These indicators not only objectively depict the socio-economic level across different regions but also possess strong representational value, providing robust data support for investigating the relationship between sports venue distribution and economic factors. Additionally, transportation conditions, a pivotal element influencing the spatial distribution of sports venues, are crucial for the accessibility to these facilities. To quantify transportation conditions, we chose the Number of Public Buses per 10,000 People (X7) and Road Network Density (X8). (2) Natural Environment Dimension: We particularly emphasized the influence of geographical terrain and climatic conditions on the distribution of sports venues. Consequently, we adopted indicators like elevation (X9), Slope (X10), and Temperature (X11) to characterize natural environmental factors. These indicators hold significant guiding significance in the planning and site selection processes of sports venues.

#### Data sources

Point of Interest (POI) Data: With the advancement of big data and its processing techniques, Points of Interest have emerged as dot-like geospatial big data representing real geographic entities. They can provide real-time descriptions of the spatial and attribute characteristics of these entities, such as name, latitude and longitude, address, type, etc. Notably, POIs are characterized by their large volume, high accuracy, and detailed classification, offering expanded possibilities for geographic spatial exploration^49,50^. In this study, we utilized the Gaode (Amap) API and employed Python web-crawling techniques to extract POI data pertaining to sports venues in China (excluding the regions of Hong Kong, Macao, and Taiwan). The data extraction was up-to-date as of July 30, 2022. After organizing, summarizing, and deduplicating the data, we employed ArcGIS 10.8 and the Geo-Detector to investigate the spatial distribution characteristics and underlying causes of sports venues in China. Additionally, the year-end total population, total GDP, per capita GDP, urbanization rate, disposable income of urban residents, temperature, and level of transportation infrastructure statistics for each province (or city/region) primarily originate from the National Statistical Yearbook, the Statistical Yearbooks of respective provinces (or cities/regions), the Sports Bureau, and the China Economic and Social Development Statistical Database. The administrative boundary vector data is sourced from the Resource and Environment Science and Data Center of the Chinese Academy of Sciences. The vector data for China’s highways come from the National Geographic Information Resources Catalog Service System’s 1:1 million basic geographic information database. The elevation vector data for China is derived from the Geospatial Data Cloud.

## Conclusions and recommendations

### Conclusions

Leveraging the Gaode (Amap) API, this study employed Python web-crawling techniques to extract POI data related to sports venues in China. Utilizing tools like ArcGIS 10.8, and integrating techniques such as kernel density estimation analysis, imbalance indices, spatial autocorrelation analysis, and the Geo-Detector, we delved into the overall distribution characteristics, regional distribution features, spatial correlations, and underlying causes of sports venue distributions across China. The primary conclusions drawn are as follows: (1) Overall Distribution Characteristics: The spatial presentation of sports venues in China predominantly exhibits a multi-centered agglomeration, an imbalanced distribution, and high-density clustering. (2) Regional Distribution Features: The spatial layout of sports venues in China demonstrates a “more in the south, less in the north” trend. Specifically, there’s a discernible pattern where the density decreases progressively from the northern coastal region, through the eastern coastal region, and to the southern coastal region, gradually dwindling towards the central regions of the Yellow River, the Yangtze River, and the northwest inland areas. (3) Spatial Correlation: The spatial distribution of sports venues roughly aligns with the “Hu-Line”, with the majority of these venues concentrated on the southeast side of this line. As observed from the Moran scatter plot, the spatial distribution of sports venues tends to follow a spatially positive correlation pattern, leaning towards high-high clustering and low-low clustering. (4) Multiple Factor Interplay: The spatial distribution of sports venues is the outcome of an interactive coupling of various factors. Based on the single-factor detection, key elements within socio-economic factors that influence the spatial distribution of sports venues encompass the overall Gross Domestic Product (GDP) and the disposable income of urban residents. The interaction detection demonstrates that the combined influence of dual factors offers a more potent explanatory power for the spatial features of sports venue distribution than individual factors alone. Hence, the spatial layout of these venues is a manifestation of the interactive coupling of a myriad of influencing factors.

### Recommendations

Based on the findings of this study, the following recommendations are put forward: (1) Differentiated Planning and Coordinated Development. Given the imbalanced distribution of sports venues across various regions, especially in the northwestern parts of China, it is imperative for the government to adopt region-specific policy strategies. These strategies should holistically consider local economic conditions, population demographics, transportation infrastructure, and environmental characteristics. A tailored approach to optimizing the structural system of sports venues will not only prevent excessive concentration of resources in specific areas but also meet the sports and recreational needs of local communities. (2) Economic Incentive Policies. The pronounced correlation between the spatial distribution of sports venues and socio-economic metrics, such as GDP and disposable income of urban residents, underscores the need for diversified incentive policies. Governments might consider offering financial subsidies, tax reductions, and discounted loan interests to mitigate the costs associated with establishing sports venues. (3) Transportation and Planning Integration. The seamless integration of sports venues with urban transportation networks is crucial. Governments are advised to increase investments in infrastructure surrounding sports venues, such as roads and public transportation, enhancing accessibility and transportation efficiency for the public. (4) Data-driven Decision-making. It is recommended for authorities to consistently harness tools like POI, ArcGIS, and Geo-detector for real-time data analytics, ensuring an up-to-date understanding of the distribution pattern of sports venues. Such continuous insights can then inform future data-backed planning and decisions for sports venue developments. (5) Environmental Consideration and Sustainability. When planning and establishing new sports facilities, it’s essential to take into account the local geographical and environmental conditions to ensure the long-term sustainability and viability of the venues.

### Limitations and constraints

Originating from a geospatial perspective, this research undertook a comprehensive exploration at the macro-level of the spatial distribution traits and causative factors of sports venues. Such analytical endeavors are pivotal for the optimal spatial arrangement of sports facilities, shedding light on their sustainable trajectory and aiding in the formulation of pertinent policies. Nonetheless, there are distinct limitations inherent in our study: (1) Our concentration predominantly lies in macro-scale examinations, leaving a vacuum in micro-scale investigations which warrant attention in subsequent research. (2) Socio-cultural backdrops and policy landscapes might also play roles in the spatial distribution patterns of sports facilities. (3) As quintessential amenities, sports venues necessitate alignment with the prevailing demographic and economic landscapes. To deepen the granularity of our comprehension, it’s recommended for future studies to augment the scope of influential determinants, incorporating methodologies like grid dimension analysis and congruence assessment models, ensuring a holistic and integrative analysis. Thus, a future-focused and in-depth approach on the aforementioned aspects is indispensable, aspiring for a strategic blueprint for sports venue evolution, accentuating holistic public health, and anchoring urban sustainability.

## Data Availability

The datasets used and/or analysed during the current study are available from the corresponding author upon reasonable request.
